# Rare case of diaphragmatic rupture following resuscitation in a pregnant woman first in literature

**DOI:** 10.1186/s13019-020-1090-9

**Published:** 2020-02-27

**Authors:** Saleem Haj-Yahia, Amro Al Aqra, Kamal Abed, Khalil Bali, Mohammad N. Sbaih, Mohanad Al Asmar, Massimo Caputo, Wafiq Othman, Ahmed Al-Adhami

**Affiliations:** 1 Cardiothoracic and Transplant Surgery, School of Clinical Sciences, Bristol Royal Infirmary, University of Bristol, Upper Maudlin StreetBS2 8HW, Bristol, UK; 2 Cardiothoracic and Transplant Surgery, Cardiovascular & Medical Sciences, British Heart Foundation, Glasgow Cardiovascular Research Centre, University of Glasgow, 126 University Place, Glasgow, G128TA UK; 3 Cardiothoracic Surgery, An-Najah National University Teaching Hospital, The National Heart and Lung Institute, Nablus, Palestine; 4 Pediatric Surgery Unit, An-Najah National University Teaching Hospital, Nablus, Palestine; 5 Faculty of Medicine, An-Najah National University, Nablus, Palestine; 6 Pediatric Cardiac Surgery, British Heart Foundation Chair, University of Bristol, Bristol, UK; 7 Cardiac Intensive Care, An-Najah National University Teaching Hospital, Nablus, Palestine; 8 Anesthesia Department, An-Najah National University Teaching Hospital, Nablus, Palestine; 9 Royal Infirmary, Edinburgh, UK

**Keywords:** Cardiopulmonary resuscitation, Diaphragmatic rupture, Pregnancy

## Abstract

**Introduction:**

Complications following Cardiopulmonary resuscitation (CPR) are rare and usually follows a vigorous CPR or in special cases like pregnancy are due to lack of knowledge and clinical practice of how to preform CPR in pregnancy. One of this complication is diaphragmatic rupture with herniation of abdominal organs. Surgical intervention needs to be planned carefully in multidisciplinary team approach and requires fine surgical techniques for better outcome**.** There are few reported cases of diaphragmatic rupture after Cardiopulmonary resuscitation but none in pregnant woman.

**Case presentation:**

We report a rare case of diaphragmatic rupture in a 29-year-old pregnant patient who experienced a full-blown diaphragmatic defect and herniation of the abdominal organs into the thoracic cavity, as a complication of CPR. Following careful assessment and diagnosis, the patient underwent urgent laparotomy with reduction of the contents and primary closure of the defect. One year follow up was satisfactory. To the best of our knowledge, this is the first reported case of diaphragmatic rupture with herniation of the abdominal organs following CPR in a pregnant woman in the literature.

**Conclusion:**

The application of external cardiac massage through CPR is a life-saving procedure for the management of cardiac arrest. Common complications related to CPR include rib fractures, sternal fractures and haemothorax. Diaphragmatic rupture with herniation of the abdominal organs is a rare complication, having been reported only once in the literature (Sabzi F, Faraji R, Tanaffos 16:170–172, 2017); however, it represents a serious and life-threating event. Thus, careful evaluation of the patient by a multidisciplinary team and prompt intervention is recommended in order to improve outcomes.

## Background

According to a recently published study, the need for cardiopulmonary resuscitation (CPR) among pregnant woman occurs in 1 in 30,000 pregnancies [1]. Cardiac arrest during pregnancy is considered to be one of the most challenging acute medical events. The difficulty arises from two main factors, the first of which is the presence of two patients, the mother and the foetus, and the second is the structural changes associated with pregnancy, specifically internal organ physiological displacement. Knowledge deficits and inadequate resuscitation skills can lead to poor outcomes for both the mother and the foetus when managing cardiac arrest.

In this case, the cause of cardiac arrest was uterine rupture, which resulted in haemodynamic and circulatory collapse. A slightly caudal mispositioned chest compressions during CPR due to the advanced stage of pregnancy led to a sudden and sharp increase in intraabdominal pressure, resulting in diaphragmatic damage and acute rupture, followed by herniation and displacement of intraabdominal organs into the chest.

## Case presentation

A 29-year-old female patient was admitted to the Gynecology and Obstetrics department at a district Southern Governmental hospital for normal delivery. The patient was later scheduled for urgent caesarean section (CS) due to foetal distress. In the operating theatre she developed sudden cardiac arrest and a full cycle of CPR as per guidelines was immediately initiated. After 5 min of effective CPR her cardiac rhythm returned to sinus rhythm, and her haemodynamic state stabilised without further use of inotropic drug support. An intra-operative general surgical team consultation was sought, and a chest CT scan was performed. She was believed to be complicated with haemopneumothorax, for which a chest drain was inserted. A CS was performed, but unfortunately, the baby died after delivery.

The patient was referred to the National Heart and Lung Institute at the university teaching hospital for further evaluation and management.

**Fig. 1:**
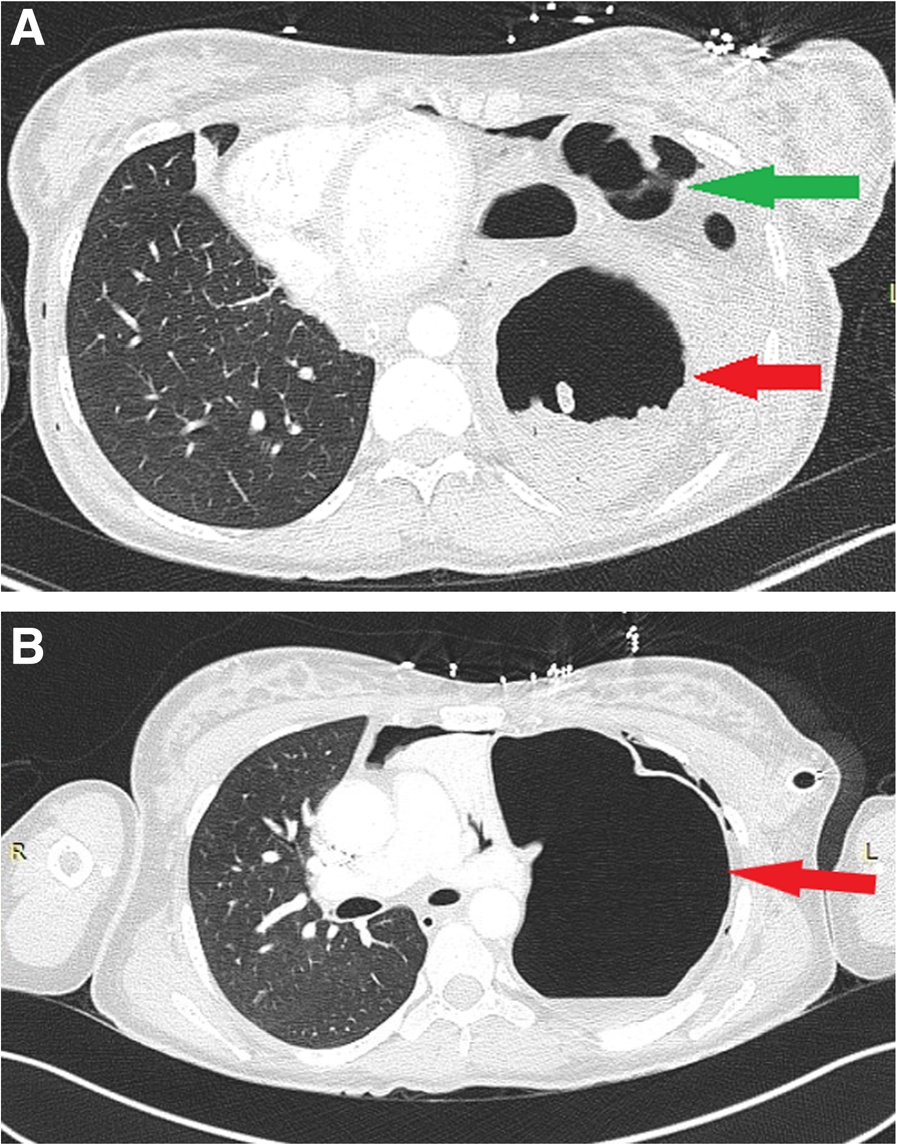
**a** and **b** Chest and Abdomen computed tomography scan (CT) showing the whole stomach (Red arrow), part of the small bowel and the colon (Green arrow) herniating into the left hemithorax

The clinical data and results of the imaging studies were reviewed and discussed by a multidisciplinary team, and the diagnosis of left-sided diaphragmatic rupture with herniation of abdominal organs through a large defect into the chest cavity was confirmed (Fig. 1a and b).

**Fig. 2:**
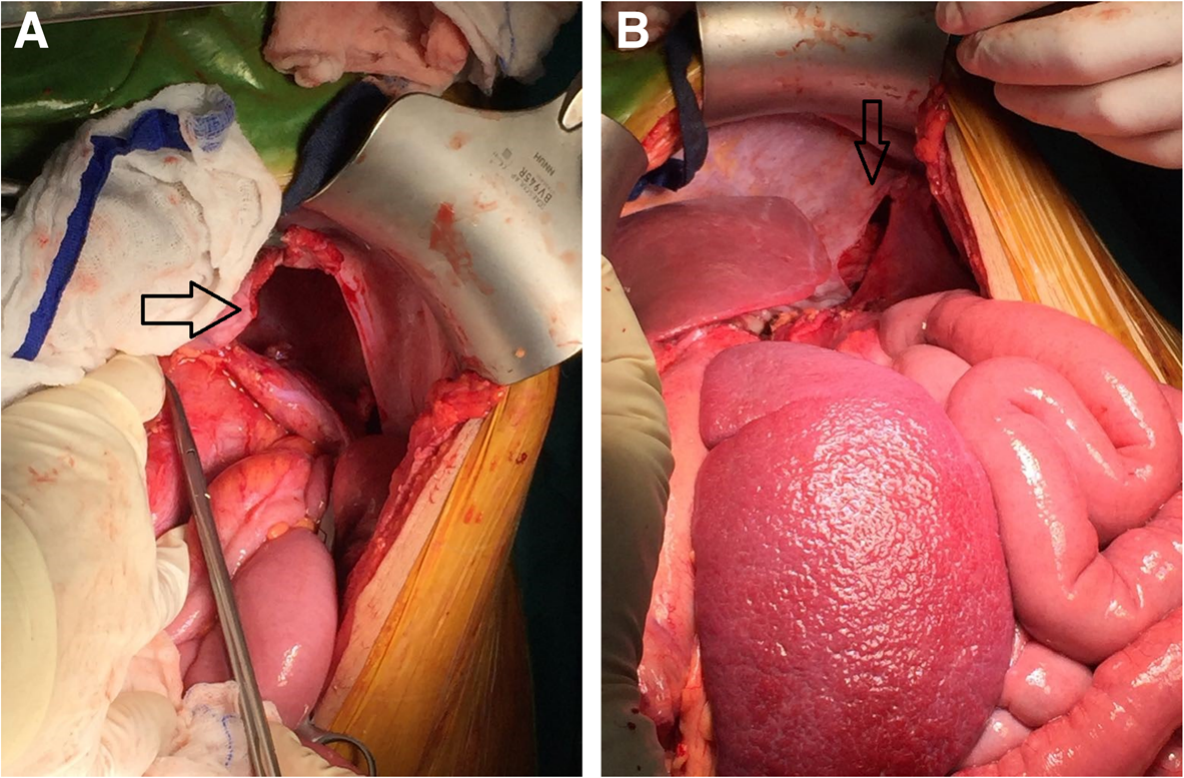
**a** and **b** Intraoperative images showing the site of diaphragmatic rupture (Black arrow) and the herniated abdominal contents

The patient was then prepared for urgent surgery and underwent subcostal open laparotomy, with intraoperative findings of a collapsed left lung, spleen, pancreas, dilated stomach, transverse colon and omentum that were displaced in the left hemithorax. Reduction of the contents was performed, and the diaphragmatic defect was repaired through primary closure with proline sutures. Lung expansion was then assisted by means of a bronchoscopy (Fig. 2a and b).

**Fig. 3:**
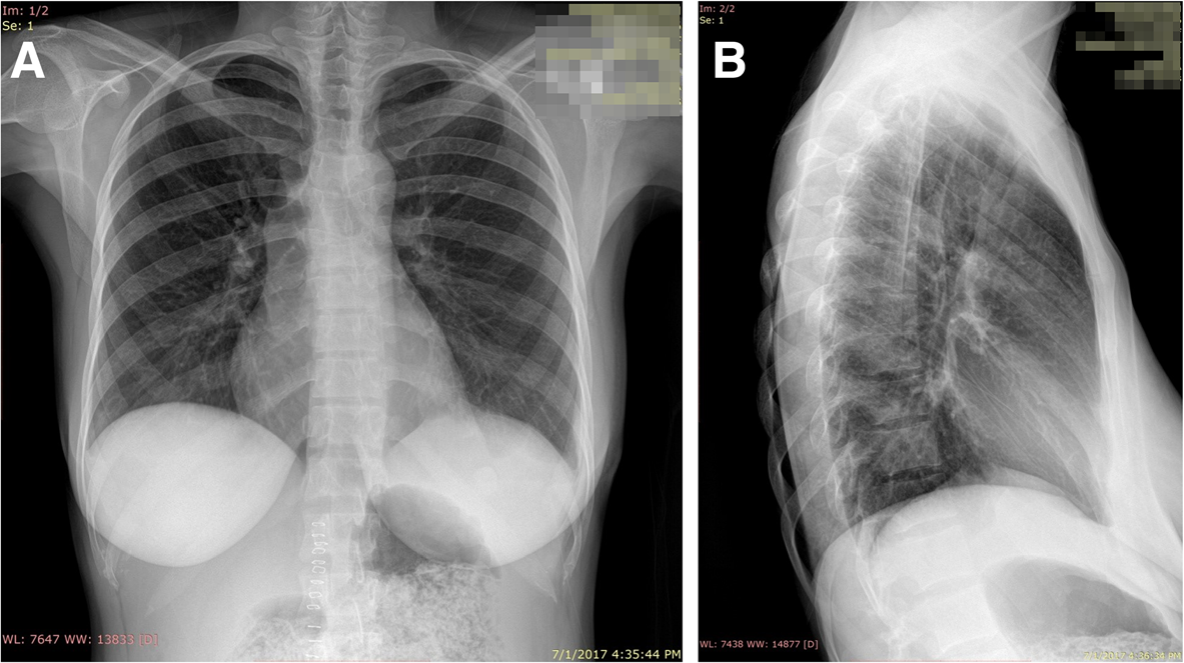
**a** and **b** One month follow up chest X-ray, completely normal

The postoperative period was uneventful, with no complications, and the patient showed excellent improvement in her clinical condition and was discharged 7 days post-operatively. At 1–month follow-up at our outpatient clinic, the patient was in excellent clinical condition with no complaints, and a chest x-ray was completely normal (Fig. 3a and b).

## Discussion and conclusion

Complications following CPR are very rare and has never been reported in pregnant patients, and usually only occur following vigorous CPR or displaced sternal compressions. In special cases, the complications are due to a lack of knowledge and clinical practice in how to perform CPR. One of these complications is diaphragmatic rupture with herniation of the abdominal organs into the thoracic cavity [2]. Surgical intervention needs to be carefully planned with a multidisciplinary team approach and requires fine surgical techniques to ensure a better outcome.

Awareness of the structural and physiological changes during pregnancy, especially in advanced pregnancy when there is significant displacement of the abdominal organs together with low compliance of the abdominal wall, is critical for the correct performance and positioning of CPR in pregnant women [3].

By reporting this case, we aim to reinforce the importance of educating medical teams in how to deal with maternal cardiac arrest in order to avoid such complications, which could be life threatening [4].

## Data Availability

Not applicable.

## Abbreviations

CPR: Cardiopulmonary resuscitation
CS: Caesarean section
